# Maintenance of flower color dimorphism in *Ophiorrhiza japonica* (Rubiaceae): responses to fluctuating temperatures in a dolomite Karst region

**DOI:** 10.3389/fpls.2024.1495112

**Published:** 2024-12-18

**Authors:** Xiao-Yue Wang, Han-Qing Tang, Yun-Jing Liu, Meng-Da Xiang, Ren-Xiu Yao, Bai-Zhu Li, Yu Li, Yin Yi, Zhi-Rui Wen, Ming Tang, Xiao-Xin Tang

**Affiliations:** ^1^ Key Laboratory of National Forestry and Grassland Administration on Biodiversity Conservation in Karst Mountainous Areas of Southwestern China, School of Life Science, Guizhou Normal University, Guiyang, Guizhou, China; ^2^ Engineering Research Center of Carbon Neutrality in Karst Areas, Guizhou Normal University, Guiyang, Guizhou, China; ^3^ School of Resources and Environmental Science, Hubei University, Wuhan, China; ^4^ School of Life Sciences, Central China Normal University, Wuhan, China; ^5^ Guizhou Collaborative Innovation Center of Green Finance and Ecological Environment Protection, Guiyang, China; ^6^ School of Chemistry, Chemical Engineering and Biotechnology, Nanyang Technological University, Singapore, Singapore

**Keywords:** flower color polymorphism, *Ophiorrhiza japonica*, pollinator mediated selection, pleiotropic effects, fluctuating temperatures, reproductive, dolomite karst

## Abstract

**Introduction:**

Flower color polymorphism is often attributed to selection pressures from Q9 pollinators or other non-pollinator stress factors. Generally, flower color polymorphism demonstrates effective acclimatization linked to either pollinator-mediated selection or pleiotropic effects.

**Methods:**

To test these hypotheses in Ophiorrhiza japonica, we compared pollinator visitation frequencies and plant traits between pink and white morphs in Shibing, a dolomite Karst region recognized as a World Natural Heritage Site. We also assessed the ratio of flower morphs and the reproductive success of the two morphs during spring and winter. Additionally, we examined the effects of temperature shifts on the two morphs under various temperature treatments.

**Results and discussion:**

Our results revealed no significant difference in visitation frequencies between the morphs. However, the ratio of pink to white morph differed significantly between spring and winter. The temperature of pink morph was higher than that of white morph at temperatures ranging from 0-24°C, while white morph had higher temperatures than pink morph at -4°C. Based on the aforementioned results, pollinators are not the primary factor influencing the distribution of flower colors in spring and winter. Furthermore, the response of different flower colors to temperature suggests that temperature is more likely the factor driving changes in flower coloration. Our study provides further evidence supporting the pleiotropic effect hypothesis, which posits that flower color polymorphism can be maintained by fluctuating temperatures in the dolomite Karst region. This study offers a potential model for explaining flower color polymorphism in Karst regions.

## Introduction

Flower color polymorphism is a relatively infrequent trait under natural conditions. It is often regarded as the result of evolutionary adaptation, determined by the identity and composition of pigments among organs, especially flowers (Rausher, 2008). Variation of flower color frequencies might represent different adaptation on a large scale of space and time in an annual plant, *Silybum marianum* (Keasar et al., 2016). According to previous studies on other species, selective pressure is usually mediated by pollinators or other non-pollinator agents (Burdon et al., 1983; Waser and Price, 1983; Brown and Clegg, 1984; Warren and Mackenzie, 2001; Coberly and Rausher, 2003; Irwin et al., 2003; Frey, 2004; Strauss and Whittall, 2006).

Variations in color polymorphism may serve as signals that attract various insects, especially pollinators (Grant, 1949; Stebbins, 1974; Melendez-Ackerman and Campbell, 1998; Hodges et al., 2002; Coberly and Rausher, 2003; Strauss and Whittall, 2006; Arista et al., 2013). Under these conditions, pollinators are generally believed to be the primary power maintaining flower color polymorphism (Baker, 1963; Stebbins, 1970; Grant, 1993). For instance, in a reciprocal transplant experiment involving *Mimulus aurantiacus*, which exhibits both red and yellow flowers, it was observed that hummingbirds preferentially selected red flowers, whereas hawkmoths showed a preference for yellow flowers (Streisfeld and Kohn, 2005). Pollinator preference for a particular flower color morph may result in greater pollen removal or pollen deposition on the stigma, leading to greater reproductive success than other morphs (Grant, 1949; Stebbins, 1974; Melendez-Ackerman and Campbell, 1998; Hodges et al., 2002). This hypothesis is known as the pollinator-mediated selection. However, not all flower color polymorphic species meet this hypothesis. For instance, *Wahlenbergia albomarginata* exhibits both blue and white morphs, with the white morph more prevalent in New Zealand than in other regions. Pollinator visit frequencies and the amount of pollen removed increased after white flowers were painted blue, raising questions about why blue morphs remain relatively rare despite their enhanced pollinator attraction (Campbell et al., 2012).

Another hypothesis is pleiotropic selection, which posits that flower color serves multiple functions, including UV protection (Koes et al., 1994), bacteriostatic properties (Gong et al., 2018), and enhanced tolerance to abiotic stress (Strauss and Whittall, 2006; Arista et al., 2013; Rathna Priya et al., 2019). In particular, flowers can repel or deter mammals, insect herbivores, or other selective agents (Lev-Yadun et al., 2018, 2024). Abiotic stress may significantly influence the maintenance of flower color polymorphisms. Pink, purple, and blue flowers are more prevalent, because anthocyanin-pigmented individuals generally exhibit greater adaptability to a range of environmental stresses. Pigmented morphs are generally more adaptive to drought (Burdon et al., 1983; Warren and Mackenzie, 2001; Tang and Huang, 2010; Rathna Priya et al., 2019) and heat stress (Coberly and Rausher, 2003) compared to white morphs. Conversely, white morphs tend to perform better under well-watered conditions or adequate spring rainfall (Warren and Mackenzie, 2001; Schemske and Bierzychudek, 2001, 2007; Tang and Huang, 2010). Temperature can exert selective pressure on flower color morphs in different ways. In *Gentiana leucomelaena*, temperature differences between blue and white flowers are influenced by color, suggesting that color variation within this species is related to temperature adaptation strategies, which may have facilitated the occurrence of flower color polymorphism (Mu et al., 2010).

Flower color polymorphisms can occur both within and among populations (Grossenbacher et al., 2021). Investigating flower color polymorphism within populations can mitigate the effects of environmental heterogeneity across different populations and provide valuable insights into the ongoing debate between the hypotheses of pollinator-mediated selection and pleiotropic effects hypotheses. In most species, flower color is often monomorphic and exhibits a uniform color within the same population. Individuals within the same population typically encounter similar selective pressures, which generally leads to trait homogeneity (Sapir et al., 2021). However, flower color can also be polymorphic and displays discrete colors in some species, as observed in *Linanthus parryae* (Schemske and Bierzychudek, 2007), *Butomus umbellatus* (Tang and Huang, 2010), and *Silene littorea* (Del Valle et al., 2019). Investigating the mechanisms that counteract this homogenization process can offer insights into how diversity is generated and why within-population variation in potentially adaptive traits is rarely maintained.

Flower color polymorphism in *Ophiorrhiza japonica* has been observed in the Shibing Dolomite Karst area, a World Natural Heritage Site in Guizhou Province, Southwest China. The term “Karst” refers to the process of rock dissolution, which results in a heterogeneous environment where interactions among plants, animals, and microbes create variable and complex ecosystems (Ford and Williams, 2007; Hartmann et al., 2014). Plants in karst regions typically adapt to drought, high temperatures, light intensity, and high calcium stress (Wei et al., 2018; Murchie and Ruban, 2019). Drought, high temperatures, and light induce changes in physiological and morphological structures that help plants adapt to karst environments. Additionally, in our previous studies, plants in karst areas adapted to pollinators (Tang et al., 2020) or extreme conditions (Tang et al., 2016) by adjusting pigment levels.

*O. japonica* exhibits pink and white morphs, and its flowering period extends from winter to the following spring, with two peak blooming phases. Unlike other plant species in Karst regions (Liu et al., 2011, 2021), *O. japonica* grows in a humid, low-light understory environments, making it less susceptible to heat, light, and drought stress. Considering the temperature variations across seasons, adaptation to fluctuating temperatures may contribute to the maintenance of flower color polymorphism in *Ophiorrhiza japonica*.

As the flowering period peaked in winter and spring, we conducted experiments during these two seasons. This study addresses two key questions: (a) Whether pollinators prefer pink or white morph, thereby testing the pollinator preference hypothesis. (b) Whether environment factors differences between the color morphs, thereby examining the pleiotropic hypothesis; Especially, it is not clear how plants perform between two morphs under different temperatures in spring and winter respectively; Therefore, we focus on whether fluctuating temperature could influence the difference between two morphs. We surveyed floral morph proportions, plant traits, and environmental conditions and tested the effects of pollinators and reproductive success under different treatments. This approach allowed us to comprehensively evaluate how flower color polymorphism may be maintained by pollinator selection and pleiotropy in the species.

## Materials and methods

### Study species and sites

*Ophiorrhiza japonica* is a hermaphroditic perennial herb (Institute of Botany, the Chinese Academy of Sciences, 1983). We observed that both red- and white-flowered individuals coexist in populations on Shibing Yuntai Mountain, located in the South China Karst World Heritage Site (26°55′13″N 108°07′47″E). Field experiments were conducted from 2016 to 2019.

### Comparison of plant traits between two morphs

To assess whether the two flower color morphs differed in traits relevant to pollination, we compared various plant traits between pink and white morphs. We measured these traits using tape measure and vernier calipers. Specifically, plant height, leaf length, and leaf width were measured with an accuracy of 1 mm using tape measure, while tube length, tube diameter, flower length, flower width, stamen length, anther length, and pistil length were measured with an accuracy of 0.01 mm using caliper micrometers. More than 30 random selected individuals of each color morph were measured. These measurements were taken in April and December 2018. Due to the complexity of the terrain, the use of the sample plot survey method proved inadequate for accurately reflecting the distribution of *O. japonica*. Therefore, we opted for a sample line survey approach. The sampling route followed the valley from the bottom to the top, beginning at the first identified location. We compared the distribution of pink and white morphs across these populations. We obtained 15 observation locations in spring(March, 2019) and 14 locations in winter(December, 2018) where we recorded the number of plants with pink and white morphs and calculated their ratio. Each *O. japonica* over 50-meter interval was recorded as a location, and all plants within a 5-meter radius of the initial population were counted.

### Pollinator behaviors and reflector spectrum

To test whether pollinators exhibited a preference between two color morphs, we conducted observations of visitor groups on pink and white color morphs. To ensure the accuracy of our results, we conducted observations at three locations with both pink and white morphs, five locations with only pink morph, and five locations with only white morph. We recorded the number of open flowers of each morph in these locations, with each location containing more than 50 individuals. Observations were made at 15-minute intervals between 10:00 a.m. and 5:00 p.m. in daytime, during periods of active foraging by pollinators in favorable weather conditions. Using a night camera (Ruiyucheng R-71) for two consecutive nights during the winter months when weather conditions are favorable. Additionally, in spring, random observations of 1 to 2 hours will be conducted each night between 7 pm to10 pm accumulating more than 5 hours cumulative to determine the presence of nocturnal pollinators. We recorded visitors as pollinators if their bodies came into contact with anthers and noted their visit numbers. Additionally, we tracked pollinators moving among individuals to quantify visit frequencies to two color morphs (visits per flower per hour). Observations were conducted during several discrete intervals: December 12–24, 2016; December 1–10, 2017; January 17–22, 2018; April 1–10, 2018; December 24, 2018–January 10, 2019; and March 19–28, 2019. The total cumulative time reaches 60 hours.

To distinguish color differences between pink and white flowers under spatial vision in insects, we employed Ocean Optics equipment to measure the reflectance spectra of both flower morphs. We recorded the diffuse spectral reflectance (300-700 nm) of individual flowers using a spectrometer (RPH-1) relative to a white reflectance standard (WS-1) under a deuterium/tungsten light source (Ocean Optics DH-2000-BAL), following the methods described by Chittka and Kevan (2005) and Ohashi et al. (2015).

### Reproductive success and breeding system

To compare reproductive success between two flower morphs, we randomly selected at least 30 healthy flowers from each treatment group for each morph to undergo bagging and performed artificial pollination when these flowers were fertile. Each flowering plant was marked according to the treatment. We then evaluated the differences in fruit set by open pollination, intra-morph pollination, and inter-morph pollination for both pink and white morphs. Open pollination was performed without any additional treatment. Intra-morph pollination involved the application of pollen from individuals of the same color morph, whereas inter-morph pollination involved the application of pollen from the opposite color morph. Flowers were bagged with mesh until they reached the female phase, and the different treatments were marked with cotton threads of various colors. Each pollination type was replicated approximately 30 times. After pollination, flowers were re-bagged to exclude pollinators. Three weeks later, we collected all labeled capsules, counted seeds, and undeveloped ovules within each capsule. These procedures were conducted in both spring (March 2018 and April 2017) and winter (December 2017 and January 2018) of both 2017 and 2018.

### Environmental factors and temperature shifts

To investigate whether color dimorphism is associated with various environmental conditions, we random measured humidity and light intensity in over 20 observing locations using a hygrometer (KIMO HD100) and a digital luxmeter (Field Scout Foot-candle Meter, Spectrum Technologies, Inc.). To standardize light measurements, we calculated the light application ratio, defined as the ratio of actual illuminance to natural illuminance in the absence of shelter. We employed a sampling survey method to locate pure white and pure red plants along the valley, from the bottom to the top. Starting at the first identified site, we recorded the color of any pure-colored flowers encountered and established a 3-meter by 3-meter plot, ensuring that all positions were spaced at least 50 meters apart. After measuring the humidity and light intensity within the plot, we counted the number of blooming flowers.

To assess the temperature differences experienced by pink and white flowers in the same wild environment, we used a thermal infrared imager (FLIR T650sc) to measure flower temperatures. FLIR T650sc is an intelligent instrument that monitors the surface temperature of objects in real time. By capturing infrared images and transferring them to a computer for professional software processing (FLIR Thermal Studio Suite 1.6.10), real-time temperature can be obtained with great accuracy, capable of sensing tiny temperature differences. Temperature is analyzed and compared in degrees Celsius.

To ensure consistency, we randomly selected pink and white flowers from mixed-morph populations. To account for environmental variability, measurements were conducted daily between 10:00 a.m. and 4:00 p.m. on several warm and cold days in both spring (March) in 2018 and winter (January) in 2019.The measurement sites refer to locations where flower color statistics are collected. Each location includes at least two flower color phenotypes simultaneously, with measurements taken hourly. During each measurement, the temperatures of the center and four sides of the flower and leaf are recorded, along with the background environmental temperature. Each season, measurements are conducted at a minimum of 10 sites for the two different flower colors.

To compare the response of the two morphs to fluctuating temperatures, we employed an artificial climate box to control environmental conditions. In 2019, we transplanted six to eight individuals from the wild into an indoor setting. The plants were cultivated indoors for several days until they were well-established and prepared for the experiment. We assessed temperature responses at -4°C, 0°C, 4°C, 8°C, 12°C, 16°C, 20°C, and 24°C using the thermal infrared imager. Each temperature was tested three times in an artificial climate box (SRG-D400B), with each trial including more than three flowers per morph.

### Statistical analysis

We compared fruit set, plant traits, and visit frequency between the two morphs using a general linear model (GLM) in SPSS version 20. Temperature between the two morphs was also assessed using SPSS version 20. Additionally, excel 2020 was used to model and calculate the effects of pollinator vision on morphs.

To assess the temperature differences experienced by pink and white flowers, we employed a general linear model (GLM) in SPSS to evaluate the effects of three variables—light application ratio, humidity, and color—on flowering quantity. Additionally, we analyzed the interaction effects among these three variables.

To examine whether pink and white morphs look different to pollinators, we compared the spectral reflectance of leaflets, petals in *O. japonica*. Eight flowers from each morph were examined at 300–700 nm range with an Ocean Optics JAZ-EL200 (Ocean Optics Inc., Dunedin, Fla.) spectrometer, with a fiber optic reflection probe (QR400-7-SR) held at 45° to the petal and leaflet surface (Johnson and Andersson, 2002).

Color patterns perceived by pollinators were represented by plotting the reflectance of the petal of *O. japonica* (pink and white morph) in a bee color hexagon model, divided into six sectors, blue (B), blue-green (BG), green (G), ultraviolet-green (UG), ultraviolet (U), and ultraviolet-blue (UB) and one uncolored central circle (Chittka, 1992), in accord with the colors perceived by bees (Backhaus and Menzel, 1987; Jersáková et al., 2006).

## Results

### Plant traits and morph proportions

**Figure 1:**
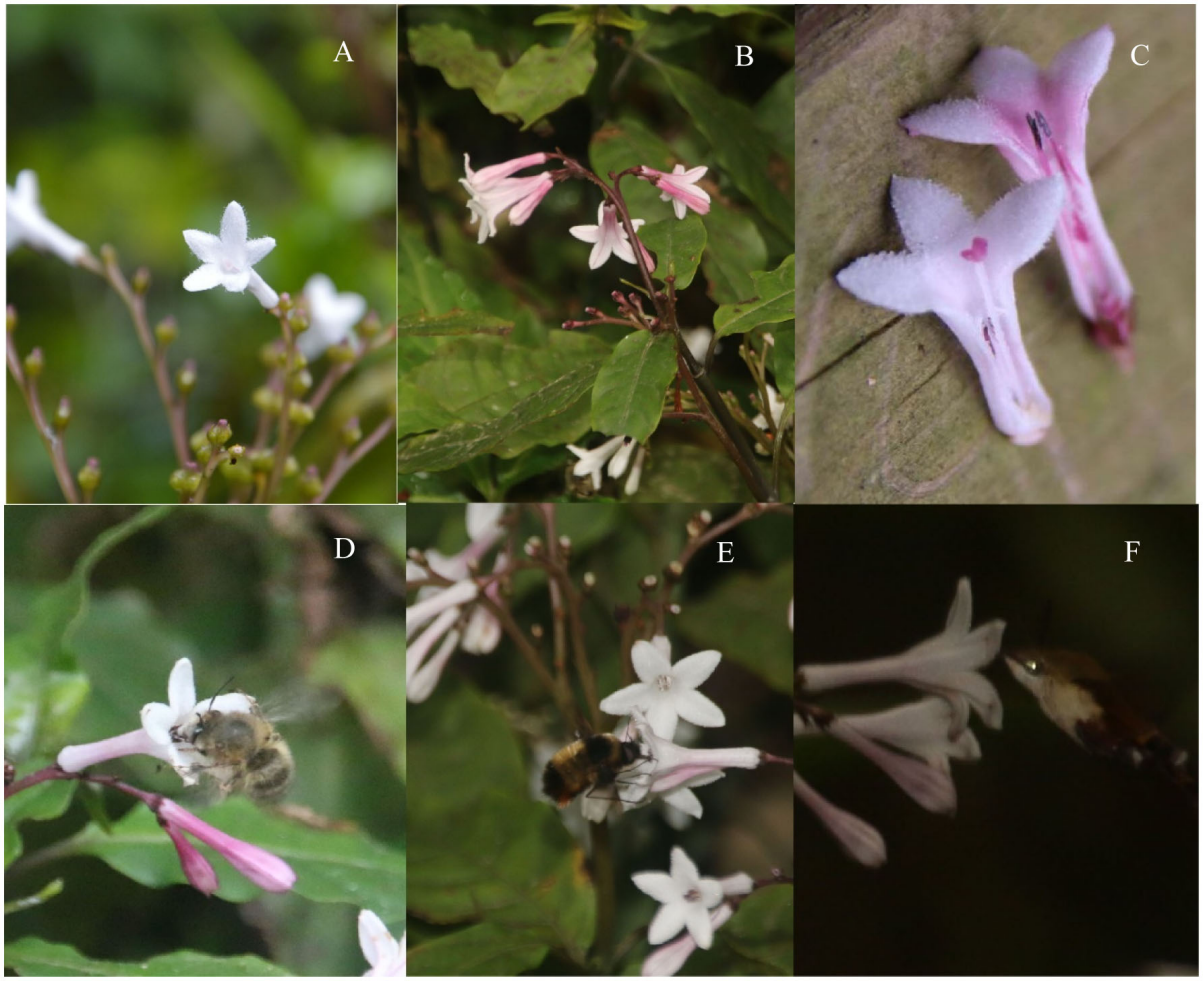
Flower color morphs and pollinators. **(A)** shows white flowers, **(B)** shows pink flowers, **(C)** illustrates the anatomy of anthers and styles, **(D)** depicts a honeybee, **(E)** shows a bumblebee, and **(F)** features a hawk moth.

Throughout the flowering period in Shibing Karst, all flowers were either pink or white (**Figure 1**). Significant differences in plant traits were observed between the two morphs. White morph individuals were taller than pink morph (P < 0.01) in winter, and their leaves were larger (P < 0.01) than those of pink morph (**Table 1**). Conversely, in spring, the traits of the pink morph were larger than those of the white morph (**Table 1**). Petal size also varied seasonally: white flowers were longer and wider in winter than pink flowers but shorter in spring.

**Table 1:** Comparing of the plant traits of *Ophiorrhiza japonica* in winter and spring.

Plant traits	Winter				Spring			
	White	Pink	Wald χ2	*P*	White	Pink	Wald χ2	*P*
Plant height (cm)	64.98 ± 2.08^a^	51.52 ± 2.70^b^	15.658	<0.01	24.69 ± 1.85^b^	35.15 ± 1.97^a^	14.988	<0.01
Leaf length (cm)	97.68 ± 3.22^a^	56.33 ± 3.82^b^	68.293	<0.01	57.36 ± 4.09^b^	83.82 ± 4.36^a^	19.695	<0.01
Leaf width (cm)	32.66 ± 1.14^a^	21.08 ± 1.02^b^	57.18	<0.01	22.76 ± 1.28^b^	29.49 ± 1.36^a^	13.034	<0.01
Flower length (mm)	9.65 ± 0.18^b^	10.70 ± 0.40^a^	5.572	<0.05	11.57 ± 0.26^b^	13.61 ± 0.27^a^	28.031	<0.01
Flower width (mm)	9.58 ± 0.23^b^	10.61 ± 0.42^a^	4.543	<0.05	11.37 ± 0.26^b^	13.22 ± 0.27^a^	24.31	<0.01
Stamen length (mm)	10.83 ± 0.68	10.22 ± 0.61	0.447	0.504	10.05 ± 0.44^b^	11.67 ± 0.46^a^	6.425	<0.01
Anther length (mm)	2.32 ± 0.08	2.29 ± 0.08	0.073	0.787	2.17 ± 0.06^b^	2.50 ± 0.06^a^	15.563	<0.01
Pistil length (mm)	10.05 ± 0.68	9.69 ± 0.61	0.328	0.567	9.54 ± 0.40^b^	11.74 ± 0.42^a^	14.394	<0.01
Tube length (mm)	11.65 ± 0.39^a^	10.55 ± 0.17^b^	6.613	<0.01	12.13 ± 0.27^b^	14.83 ± 0.29^a^	46.072	<0.01
Tube diameter (mm)	4.40 ± 0.16^a^	3.20 ± 0.17^b^	36.944	<0.01	3.17 ± 0.07	3.08 ± 0.07	0.821	0.365

Different letters indicate significant differences.

**Figure 2:**
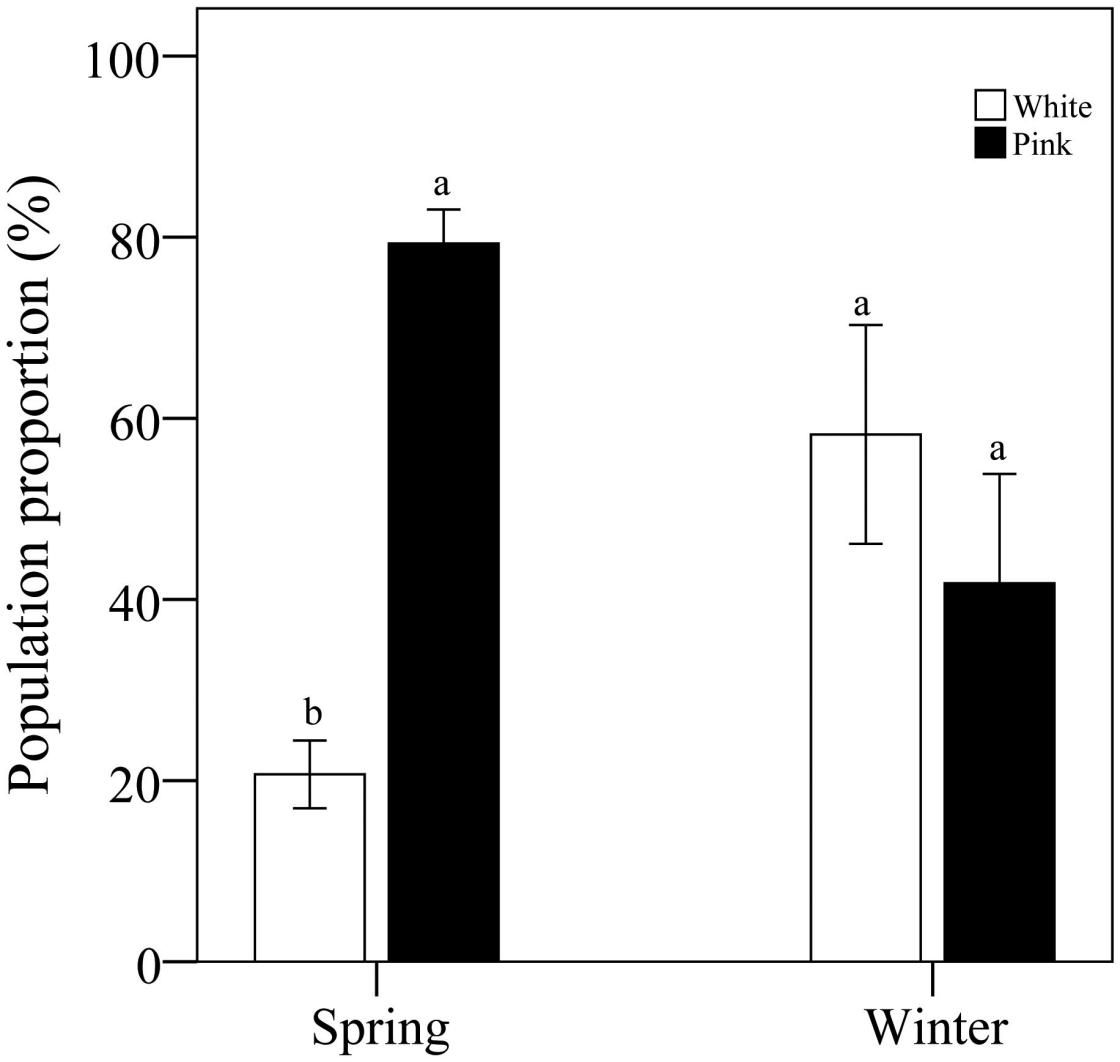
Comparative analysis of color morph percentage in pink and white flowers under various conditions. Different letters indicate significant differences.

Analysis of color morph ratios revealed that, in spring, data from 15 locations and 3,775 flowers indicated that the proportion of pink morphs was 41.4% higher than that of white morphs. In winter, data from 14 locations and 255 flowers showed that the proportion of white morphs was 39.3% higher than that of pink morphs (**Figure 2**).

### Pollinator behaviors and reflector spectrum

**Figure 3:**
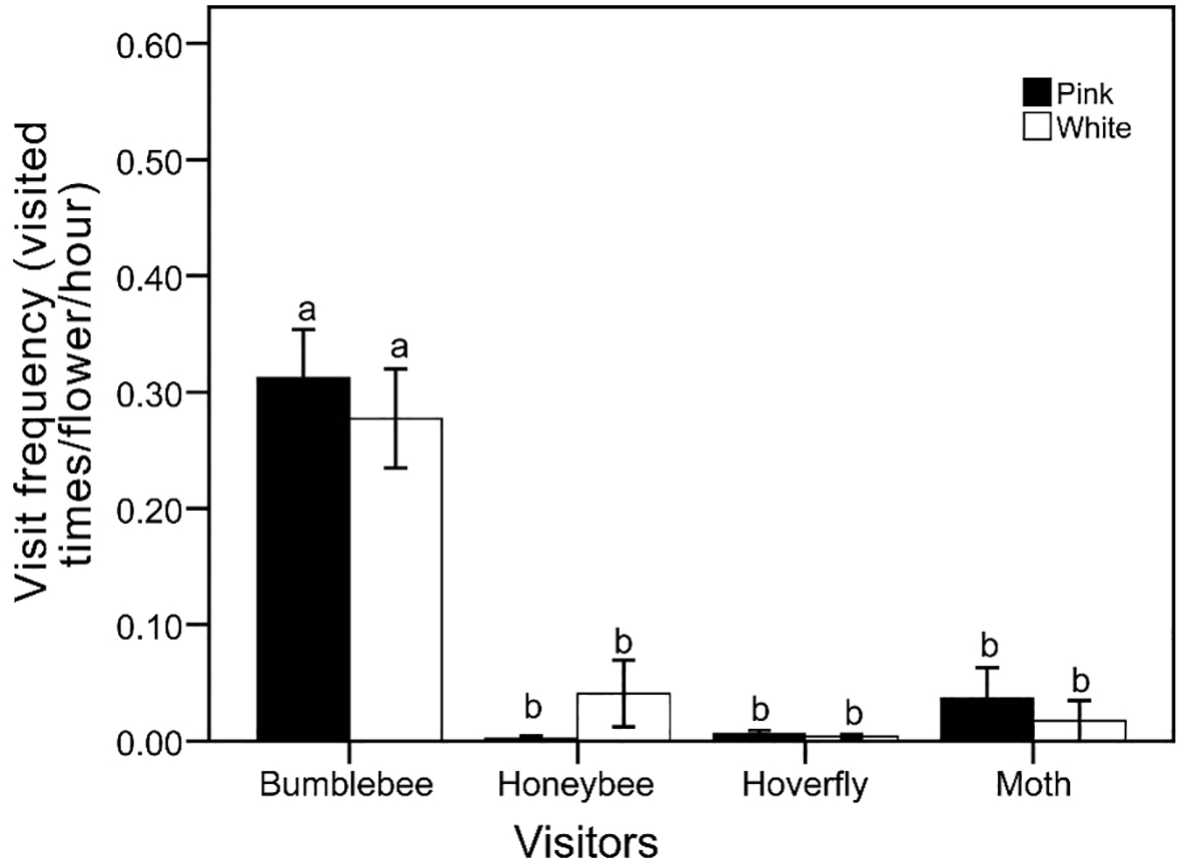
Comparing visitor frequencies between two flower color variants. Significant differences indicated by different letters.

**Figure 4:**
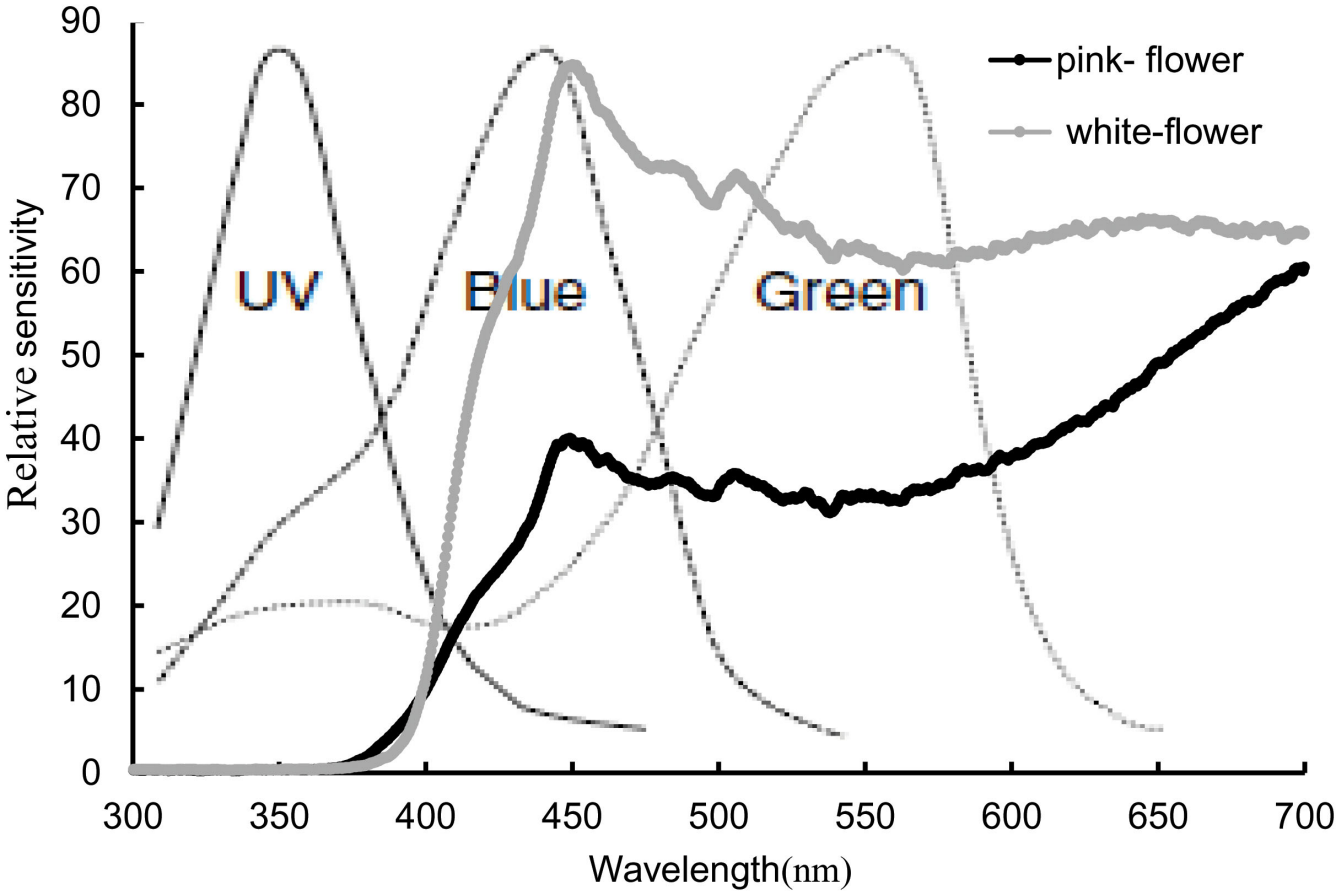
Spectral reflectance of pink and white flowers and their relative sensitivity to bee vision. The Dotted line represents the relative sensitivity of bee visual.

**Figure 5:**
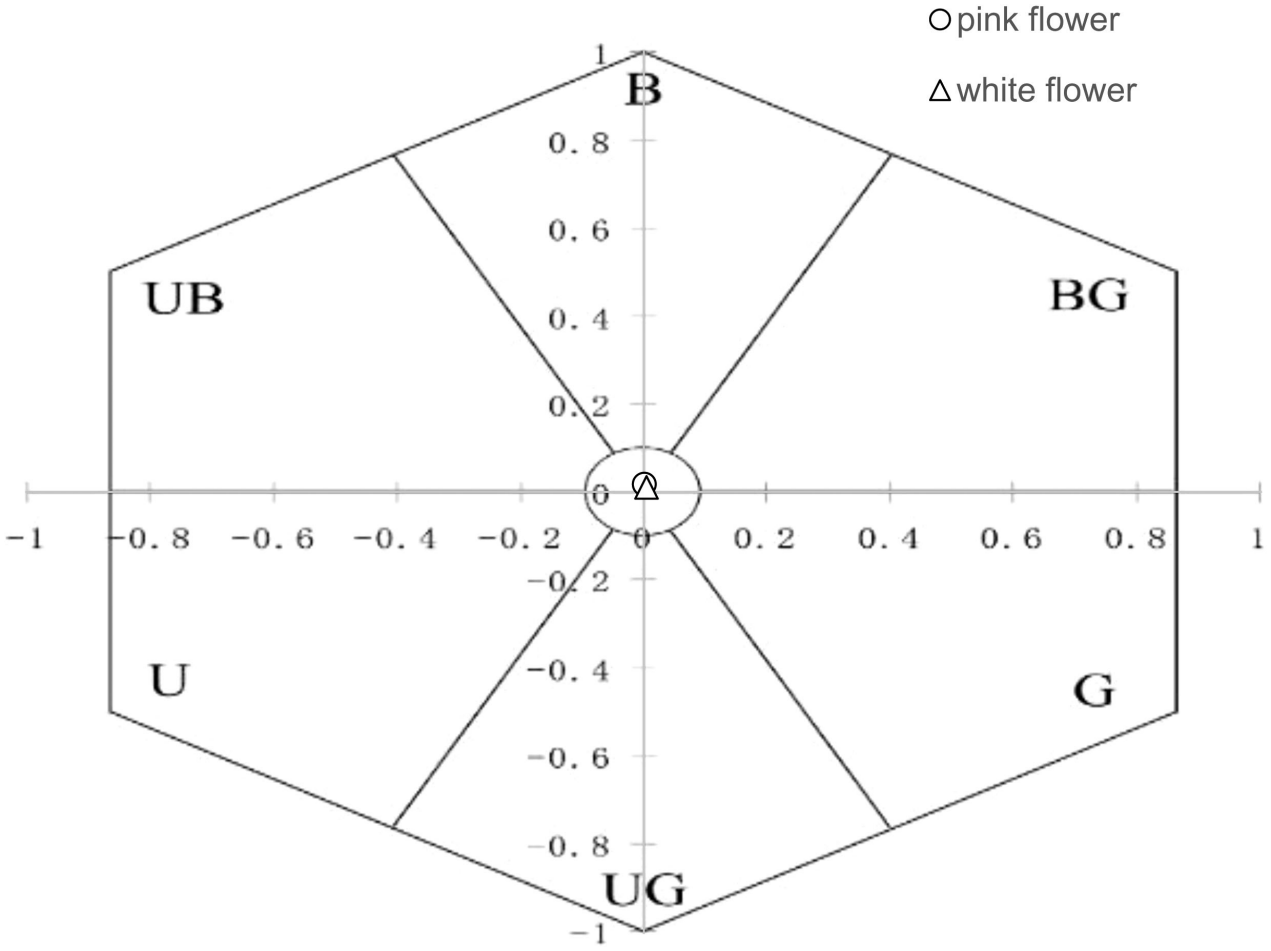
Color perception of white and pink flowers against a leaf background in bee vision.

We observed visitor activitiesbetween the two flower color morphs during both winter and spring. In winter, visitor activity was minimal in daytime, with no visitors recorded during nighttime observations. In spring, bumblebees were the primary visitors to both pink (0.312 ± 0.04 visit/hour/flower) and white (0.278 ± 0.04 visit/hour/flower) morphs, and no significant difference in visit frequency was found between the two morphs. Honeybees (pink, 0.002 ± 0.02 visit/hour/flower; white, 0.041 ± 0.03 visit/hour/flower) and hoverflies (pink, 0.006 ± 0.03 visit/hour/flower; white, 0.004 ± 0.02 visit/hour/flower) were observed infrequently. Moths were seen with a high frequency of visits and rapid movement and visitation during the day; however, their overall occurrence was rare, with low total frequencies (pink, 0.037 ± 0.03 visit/hour/flower; white, 0.017 ± 0.02 visit/hour/flower). No significant difference (P > 0.05) in visit frequency was observed between the two morphs (**Figure 3**). The reflectance in the blue and green spectra differed markedly between the two morphs (**Figure 4**). We assessed the ability of bees to recognize white and pink flowers against leaf backgrounds. The results indicated that both flower morphs were poorly distinguishable, with no significant difference in detectability by bees (**Figure 5**).

### Seasonal variations in reproductive performance across flower color morphs

**Figure 6:**
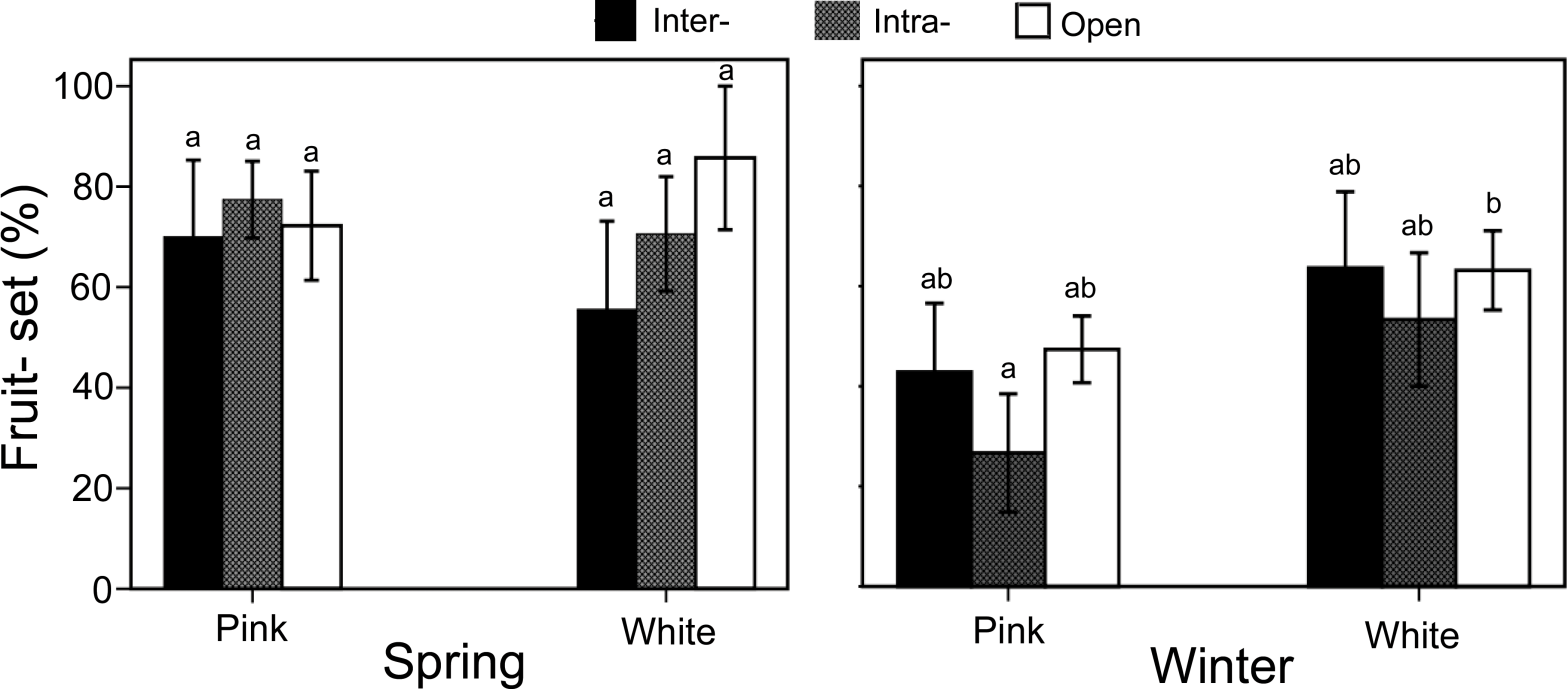
Fruit set in Ophiorrhiza japonica across two morphs during spring and winter. Different letters indicate significant differences.

In winter, the fruit set percentages for the three pollination treatments in white morphs were as follows: inter-morph pollination (63.6 ± 15.2%), intra-morph pollination (53.3 ± 13.3%), and open pollination (63.2 ± 8.0%). No significant differences were observed between the treatments. For pink morphs in winter, the fruit sets were: inter-morph pollination (42.9 ± 13.7%), intra-morph pollination (26.7 ± 11.8%), and open pollination (47.4 ± 6.7%). Similarly, no significant differences were found between the treatments (P > 0.05). Additionally, within the same season, there were no significant differences in fruit set between white and pink morphs for any treatment (**Figure 6**).

In spring, the fruit sets for the three pollination treatments in white morphs were: inter-morph pollination (55.6 ± 17.6%), intra-morph pollination (70.6 ± 11.4%), and open pollination (85.7 ± 14.3%). No significant differences were detected between the treatments. For pink morphs in spring, the fruit set percentages were: inter-morph pollination (70.0 ± 15.2%), intra-morph pollination (77.4 ± 7.6%), and open pollination (72.2 ± 10.9%). No significant differences were observed between the treatments. Similarly, within the same season, there were no significant differences in fruit set between the white and pink morphs for any treatment (**Figure 6**).

### Environmental factors and fluctuating temperature

**Figure 7:**
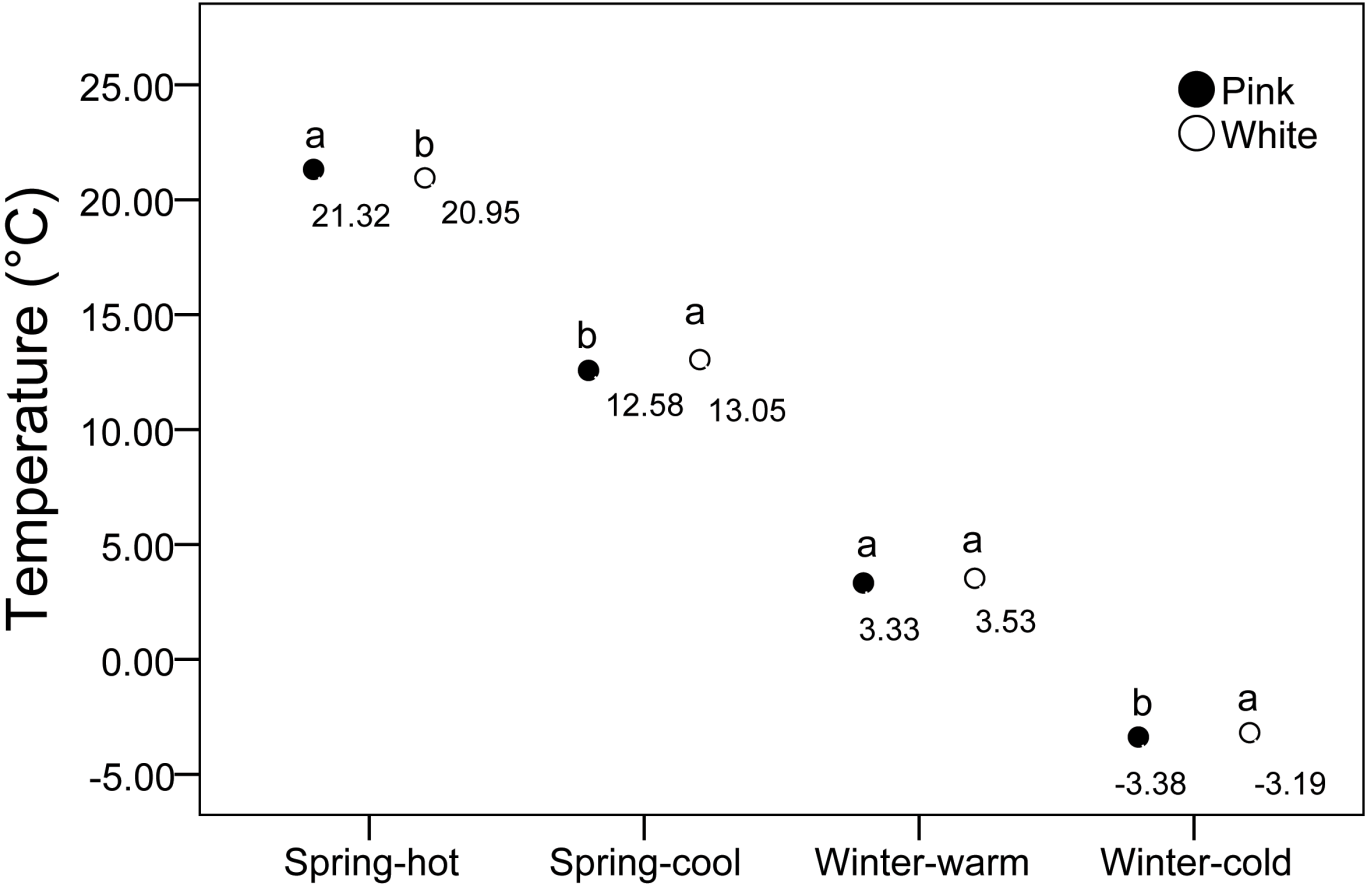
Temperature variations in response to different temperature gradients between two morphs.

**Figure 8:**
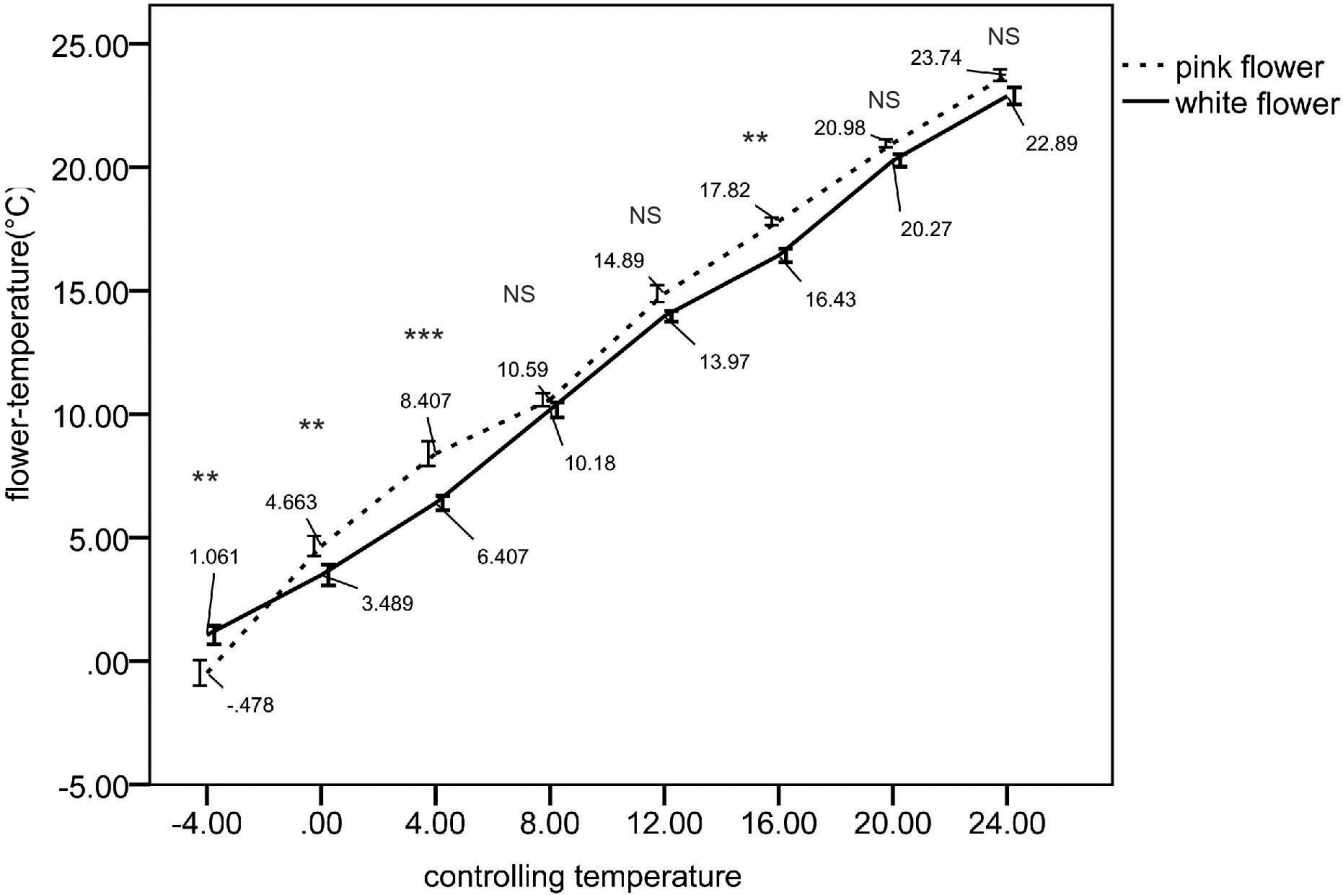
Temperature shifts on floral surface across different color morphs under a temperature gradient.

We assessed various environmental factors for the two color morphs at 20 measurement locations. The average illuminance in biotopes without shelter was 37.95 ± 1.92 lux, compared to 11.29 ± 0.84 lux in biotopes with vegetation. Given the significant variation in illumination due to weather conditions, we examined the correlation between light application ratios and color polymorphism, which was found to be low (**Table 2**). The average humidity ranged from 80% to 90% and showed a low correlation with color polymorphism (**Table 2**). Under high-temperature conditions, pink flowers were warmer than white flowers, whereas under low-temperature conditions, white flowers were warmer than pink flowers (**Figure 7**). These results suggest that pink flowers exhibit higher activity at elevated temperatures and lower activity at cooler temperatures than white flowers. We also measured temperature differences between the two morphs across various temperature gradients. At -4°C, pink flowers were cooler than white flowers, but from 0°C to 20°C, pink flowers were warmer than white flowers (**Figure 8**).

**Table 2:** Interaction effects of light application ratio, humidity and color on the blooming flowers of *Ophiorrhiza japonica*.

Condition	Wald χ^2^	df	*P*
Flower color	1.968	1	0.161
Light application ratio	1.063	3	0.786
Humidity	1.004	1	0.316
Flower color * light application ratio	1.095	1	0.295
Flower color * humidity	0.219	1	0.640
Light application ratio * humidity	0.040	1	0.841
Flower color * light application ratio * humidity	0.040	1	0.841

* represents an interaction effect.

## Discussion

### Pollinator mediated selection on flower color dimorphism

Flexibility in pollinator color preferences can significantly affect the evolution of floral traits. Variations in pollinator preferences result in increased floral visitation, which in turn drives selection for favored flower types (Waser and Price, 1981; Levin and Brack, 1995; Schemske and Bradshaw, 1999; Aldridge and Campbell, 2007).

Previous research has revealed that pollinator-mediated selection is unexpectedly limited (Trunschke et al., 2021). In a phenotypic selection study, leafcutting bees were found to select flowers based on hue or flower color, whereas bumblebees chose flowers based on chroma or darkness (Brunet et al., 2021). Wassink and Caruso (2013) observed significant net selection for color saturation in *Lobelia siphilitica*. Flower pigment significantly influenced both pollinator visitation and resistance to seed predators, resulting in substantial net selection of flower color (Carlson and Holsinger, 2012; Veiga et al., 2015). In a population of *Iris pumilla*, a significant increase in anthocyanin concentration was detected in the blue-flowered morph, whereas no such selection was observed in the purple morph (Souto-Vilarósa et al., 2018).

In our study, neither the frequency of visitors nor the visual perception of bees showed significant differences between the two color morphs, and there were significant changes in the morphological traits of the flowers. This suggests that color alone is unlikely to influence visitor preference between the two morphs. Additionally, fruit set data indicated that flower color did not affect reproductive success. Thus, color polymorphism in *Ophiorrhiza japonica* may not be an adaptation to pollinators. However, the number of pink morphs was greater than that of the white morphs. We also investigated the interaction between flower color and light application ratio but found no significant effect. In contrast to our previous studies on other species in the Karst region, which indicate that flower color dimorphism may be maintained by pollinator-mediated selection, this study examined the case of *Allium wallichii* (Tang et al., 2020). Therefore, the potential role of pollinators in maintaining color polymorphism in other regions should be carefully considered.

### Maintenance of flower color dimorphism by abiotic environmental factors

Many enzymes involved in anthocyanin synthesis are crucial for the production of other flavonoid compounds. These compounds affect not only flower color but also various ecological and physiological traits in plants (Shirley, 1996). Consequently, several researchers have proposed that the evolution of flower color may be influenced more by the selection on these pleiotropic effects than by pollinator-driven selection, or that both factors may contribute (Rausher and Fry, 1993; Simms and Bucher, 1996; Fineblum and Rausher, 1997; Armbruster, 2002; Irwin et al., 2003).

Furthermore, pigments responsible for flower color may play a role in resistance to abiotic stressors, such as high temperatures, low precipitation, high ultraviolet radiation, and low productivity environments (Schemske and Bierzychudek, 2001; Chalker-Scott, 2008; Arista et al., 2013; Koski and Ashman, 2015). Flavonoid development can be influenced by temperature (Ben-Tal and King, 1997; Jaakola and Hohtola, 2010). Evidence indicates that temperature can differentially impact the fitness of flower morphs with low versus high color saturation (Coberly and Rausher, 2003, 2008). If anthocyanins provide heat stress resistance, then higher temperatures are expected to favor flowers with greater chromatic contrast than those at lower temperatures.

*Ophiorrhiza japonica* exhibits a prolonged flowering period that spans both spring and winter, and is susceptible to significant temperature fluctuations. Observations indicate that pink flowers are warmer than white flowers at high temperatures, but cooler at low temperatures, suggesting that pink flowers are more active under warmer conditions. Temperature tests revealed that the temperature response between the two morphs varied across different temperature gradients, implying that temperature may influence flower color polymorphism. Generally, plant temperature reflects its activity level (Kim et al., 2016). Within the 0-20°C range, pink morphs demonstrated higher activity compared to white morphs, while white morphs exhibited greater activity at -4°C. No significant differences were observed at temperatures greater than 24°C. In spring, most characteristics of pink individuals are significantly larger than those of white individuals; however, in winter, the trend reversed, suggesting that pink morphs might possess an adaptive advantage to heat. These findings indicate that flower color in *O. japonica* is associated with heat stress, with pink morphs showing better fitness under such conditions. Thus, flower color polymorphism may be primarily associated with heat stress rather than honeybee preference or other factors affecting color and morphology. These findings align with those of studies on *Gentiana leucomelaena*, which exhibited different morphs adapted to varying temperature conditions (Mu et al., 2010). Our previous research also supports these results, demonstrating that temperature also plays a crucial role in maintaining flower color polymorphism in *Geranium nepalense* (Tang et al., 2016).

Coberly and Rausher (2003) demonstrated that flower color, regulated by the flavone gene, can alleviate heat stress. High temperature negatively impacts the fertilization success of both male and female homozygous *Ipomoea purpurea* individuals. Additionally, individuals with the white morph, resulting from a loss-of-function mutation, exhibit reduced fertilization success at high temperatures compared with pigmented individuals, although this effect is not observed at low temperatures. These results align with observations and further support the role of flavones in regulating flower color in *Ophiorrhiza japonica* (e.g. Sun et al., 2019, 2021).

The MADS-box transcription factor FLOWERING LOCUS M (FLM) has been implicated in phenotypic variation related to plant growth and color (Lee et al., 2013). Leaf temperature in *flm-3* mutant plants was lower than that in wild-type plants. To quantify the adaptive effects of FLM alleles in natural environments, common garden experiments should be conducted at locations with varying temperature fluctuations and precipitation levels (Ågren and Schemske, 2012). FLM is a well-known transcription factor whose splicing variation in response to temperature change modulates flowering time (Scortecci et al., 2001; Balasubramanian et al., 2006). In the present study, the pink morph produced more individuals than the white morph in spring, whereas the white morph produced more individuals than the pink morph in winter. At higher temperatures, pink flowers were warmer than white flowers, while white flowers were warmer than pink flowers under low-temperature conditions. Thus, the pink morph is more adaptive to warmer weather conditions, whereas the white morph is better suited to cooler conditions.

## Conclusion

Flower color polymorphism is usually considered an adaptation process to environmental stress. Two hypotheses, pollinator-mediated selection and pleiotropic effects, have been proposed to explain this phenomenon, which means that either pollinators or other non-pollinator factors (such as herbivores or abiotic stresses) could contribute to the evolution of flower color polymorphism. Our study provides further evidence supporting the hypothesis that flower color polymorphism of *O. japonica* can be maintained by fluctuating temperatures in Shibing, a dolomite Karst area of the World Natural Heritage Site.

Although pollinator-mediated selection did not significantly affect flower color polymorphism in *Ophiorrhiza japonica* in this area, its potential role in maintaining color polymorphism in other species or Karst regions should not be disregarded. Our previous studies have demonstrated that flower color dimorphism may be maintained by pollinator-mediated selection in *Allium wallichii*. It is possible that a diversity of pollinators exists within small areas because of the complex habitats and diverse microenvironments in Karst region. The interaction between pollinator effects and other abiotic or biotic stresses remains unclear, including whether these factors promote or counteract each other in influencing flower color polymorphism.

This study offers a potential model to explain flower color polymorphism. Further research should focus on additional examples of color polymorphic species and integrate both morphological and molecular evidence to elucidate how flower color polymorphism is maintained in Karst regions.

## Data Availability

The raw data supporting the conclusions of this article will be made available by the authors, without undue reservation.
